# Effects of Monopolar Dielectric Radiofrequency Signals on the Symptoms of Fibromyalgia: A Single-Blind Randomized Controlled Trial

**DOI:** 10.3390/ijerph17072465

**Published:** 2020-04-03

**Authors:** Alfonso Javier Ibáñez-Vera, Jerónimo Carmelo García-Romero, José Ramón Alvero-Cruz, Rafael Lomas-Vega

**Affiliations:** 1 Department of Health Sciences, University of Jaén, 23071 Jaén, Spain; 2 Department of Human Physiology, Human Histology, Pathological Anatomy and Physical Education and Sports, University of Málaga, 29071 Andalucía TECH, Spain

**Keywords:** electromagnetic radiation, fibromyalgia, quality of life, pain

## Abstract

Monopolar dielectric radiofrequency (MDR) is a non-invasive treatment for pain based on the local application of electromagnetic signals. The study’s goal was to analyze the effects of MDR on the symptoms of fibromyalgia. For this aim, a randomized controlled trial was conducted on 66 female participants (aged 47 ± 17.7) diagnosed with fibromyalgia. Participants were randomly allocated to either an experimental group (*n* = 23), which received eight 20-minute sessions of MDR; a sham group, which received the same number of sessions of a sham MDR therapy (*n* = 22); or a control group (*n* = 21), which received usual care. The outcome variables included pain measured by the visual analogue scale (VAS), score on the hospital anxiety and depression scale (HADS) and quality of life measured by the combined index of fibromyalgia severity (ICAF). A large effect size was observed for the local pain (R^2^ = 0.46), total ICAF (R^2^ = 0.42) and ICAF physical factor scores (R^2^ = 0.38). Significant mean differences were found for the local pain (*p* = 0.025) and ICAF physical factor (*p* = 0.031) scores of the experimental group in comparison with the sham group. No statistically significant differences between groups were found in HADS. In conclusion, MDR is more effective than either sham treatment or usual care in the short-term improvement of pain and the physical wellbeing of participants with fibromyalgia.

## 1. Introduction

Fibromyalgia is a syndrome that has a dramatic impact on the quality of life of patients [1]. This syndrome is endemic to developed countries, where it presents a 2.1% rate of prevalence and has risen remarkably in the last years [1]. The aetiology of fibromyalgia still remains unknown, although several hypotheses exist. On the one hand, this disorder is associated with hyporeactivity caused by exhaustion of the hypothalamic–pituitary–adrenal axis, which influences stress, metabolism, and the immune system [2]. However, a study performed by Albrecht et al. suggests that its origin may be found in blood flow deregulation as a result of excessive innervation to arteriole–venule shunts [3]. The most recently formulated hypothesis concerns small-fiber neuropathy, a condition that 49%–63% of fibromyalgia patients exhibit, as well as large fiber-demyelinating and/or axonal sensory-motor polyneuropathy, presented by 90% of fibromyalgia patients [4,5]. In spite of this, the most widely accepted hypothesis concerns central sensitization, a disorder related to pain-induced changes in the brain that result in increased pain sensitivity [6].

Although its main manifestation is diffuse pain throughout the whole body [7], other related symptoms may appear such as muscle stiffness, depressive impairment, digestive alterations, fatigue, lack of restful sleep, headache, irritability, and thermal sensitivity [8]. As an example, the cost of fibromyalgia in Spain has been estimated by the Spanish Rheumatology Association at € 7.813 per year and patient [9]. This cost becomes even more significant if we consider that treatment for this syndrome is merely symptomatic. These costs include medical consultation, pharmacological and physical therapy treatment, as well as other treatment fees not covered by national public health services, and which are therefore paid by patients directly [10].

As already seen, fibromyalgia is much more than pain. Nevertheless, the relationship among pain and other outcomes, such as quality of life or mood, means the treatment of fibromyalgia is based on a symptomatic approach using analgesic drugs, opioids, anticonvulsants such as pregabalin or gabapentin, and antidepressants such as milnacipran, amitriptyline, and duloxetine [11,12,13]. Because of the modest therapeutic results obtained and the tolerance and dependency risks that some of these drugs may present, several revisions have pointed out the need for complementing treatment with physiotherapy and physical exercise [14,15]. To that end, various approaches have been devised to apply physical therapy to the relief of fibromyalgia symptoms. In this regard, the application of manual therapy is still based on poor evidence and has shown poor-to-moderate results as far as pain relief and the quality of life of patients are concerned [15]. On the matter of therapeutic physical exercise, several aerobic and strength training programs have been reported to be effective in relieving pain and improving the quality of life of patients [16,17]. Regarding physical techniques, the quality of evidence available is even scarcer. Within this field, transcutaneous electrical nerve stimulation (TENS) is the most studied non-invasive procedure which seems to be effective for pain treatment, quality of life and depression improvements in the short term [18,19]. This technique consists of the application of two adhesive electrodes on the skin that transmit low frequency electric stimulation to reduce pain by the control gate mechanism [18,19,20]. However, other non-invasive techniques seem effective in pain treatment, such as dielectric transmission radiofrequency [21]. This procedure allows energy to be transferred non-invasively to deeper tissues than with conventional electrotherapy or thermotherapy systems [22]. Additionally, this system profits from tissue dielectric charge capacity [23] to deeply transport high-frequency energy while avoiding warming the surface or any metallic material. At the same time, it allows a higher degree of focalization and density of energy applied [22]. This and similar systems may improve the effectiveness of electrotherapy and act as an analgesic therapy for patients in chronic pain, as their adverse effects are small in comparison with those of medication [12,24]. 

Due to the dependency risk of analgesic drugs and the poor effects of other complementary approaches, the main objective of this paper is to analyze the effects of MDR for symptomatic treatment in patients with fibromyalgia regarding pain relief and quality-of-life improvement. 

## 2. Materials and Methods 

### 2.1. Design

This study was approved by the Committee of Research Ethics of the Andalusian Health Service (Málaga), with clinical trial registration number ACTRN126170001499370, and was performed according to the Helsinki Declaration, good clinical practice rules, and all applicable laws and regulations. All participants signed a written informed consent form. 

Sample size calculation was performed using the statistical software ENE 3.0 (Servei d’Estadistica Universitat Autónoma de Barcelona, Barcelona, Spain). Data provided by Sutbeyaz et al. [25] were taken as the basis for finding differences between two samples. With a significance level of *p* ≤ 0.01 and a statistical power of 90%, 13 participants per group were required. Taking into consideration an expected loss rate of 12.5% [25], at least 15 participants were required per group. This amounts to a minimum of 45 participants for this study. 

**Figure 1:**
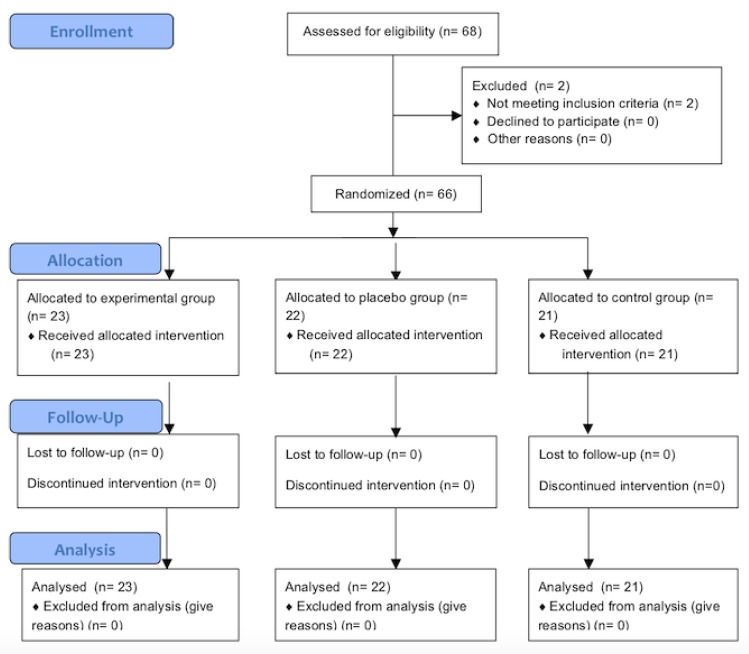
Flow diagram depicting flow of participants through each stage of the trial.

To increase the power of the study, the randomized controlled trial was carried out on a population of 68 eligible female participants. These were chosen from among members of two associations of fibromyalgia patients based in two towns in the province of Málaga, Spain. Sixty-six participants over 18 years old, which had been previously diagnosed with fibromyalgia according to the criteria of the American College of Rheumatology (2010), were included in the study [8]. These criteria have shown a high degree of coincidence with the originals of 1990 [26]. Participants underwent their usual pharmacological treatment throughout the whole study so as to minimize changes. Participants were excluded if they took part in other studies, had changed their medication in the last three months or at any time through the duration of this study, used pacemakers or drug perfusion pumps, were subject to anti-tumoral treatment at any time in the last two years, suffered from thermal sensitivity alterations, received other treatments such as physiotherapy or exercise through the study, or thought they might be pregnant. Participants were randomly assigned to one of the three groups (control, sham, or experimental) using opaque envelopes that were distributed by one of the researchers who was blinded to their content (Figure 1). 

### 2.2. Intervention

**Figure 2:**
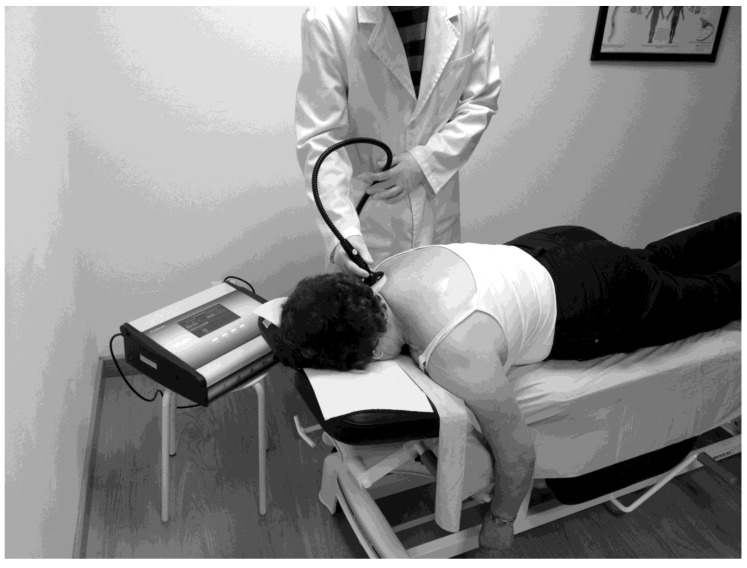
Application of monopolar dielectric electromagnetic signals (MDR) on the upper trapezius region.

The experimental group received eight 20-minute sessions of therapy using MDR according to the procedure of a previous study [21]. Sessions took place from Monday (beginning just after the baseline measures) to Friday in the first week, and on Monday, Wednesday, and Friday in the second, final measures being collected just after the last session of treatment. All sessions took place in an ambulatory facility in Málaga, under the clinical conditions required by current laws and regulations. The emission had a frequency of 870 kHZ, and an intensity of 30A in pulsed emission of 50% so as to reduce thermal impact. This therapy was applied using a non-invasive radiofrequency device for pain treatment (Physicalm®, Biotronic Advance Develops®, class IIa device, Granada, Spain). The upper trapezius was the area chosen for application as this is where subjects experienced pain most frequently (Figure 2). 

Regarding the sham group, they received the same protocol as the experimental group but with a non-emitting device whose software and hardware were apparently the same. As for the control group, they continued receiving their usual care and maintained their usual physical activity level as well as their pharmacological and physical treatment plan.

### 2.3. Outcome Measures

Before the intervention, an assessor evaluated participants using validated questionnaires on pain and quality of life. The same person was responsible for carrying out measurements of age, weight, height, years diagnosed and body mass index. This assessor was blind to assigned intervention, and absent when the envelopes were opened and treatment was applied. 

#### 2.3.1. Pain

Pain was measured using the visual analogue scale (VAS). This is a 10cm line marked “no pain” (0 value) in one end and “the worst pain which can be imagined” (value 10) on the other [27]. VAS has shown an intraclass correlation coefficient (ICC) of 0.97, a standard error of measurement (SEM) of 0.03 and a minimum detectable change (MDC) of 0.08 in patients with musculoskeletal problems [28]. Individuals are asked to draw a perpendicular line at some point between both ends according to the pain intensity they perceived in their upper trapezius region, and also generally in the whole body. Their perceived pain was thus assessed before treatment and just after the intervention. 

#### 2.3.2. Quality of Life

Quality of life was measured using the ICAF (combined index of fibromyalgia impact in patients). This scale has been validated for a Spanish population of fibromyalgia patients showing a ICC of 0.86 and a SEM of 0.20 [29]. In it, four factors are considered: a physical factor or PF (subdivided into five categories: “intensity of pain”, “sleep quality”, “impact”, “fatigue”, and “functional capacity”), an emotional factor or EF (containing “anxiety and depression” and “general health”), an active coping (AC) factor (divided into “active coping strategies” and “self-efficacy”) and finally a passive coping factor or PC (containing only the “passive coping strategies” category). The result of adding the scores for the physical, emotional and passive coping factors is then multiplied by a coefficient previously determined by the questionnaire. From this number we subtract the score of the active coping factor multiplied by the corresponding coefficient. The result represents the level of severity a patient experiences. Higher final scores reveal a more severe condition. This questionnaire was applied before the first treatment session and again after the end of the last one.

#### 2.3.3. Mood 

Participants’ mood, signs of depression and anxiety levels were measured using the hospital anxiety and depression scale (HADS) [30]. This is a Likert-type scale that contains 14 questions concerning participants’ sensations and mood. Regarding to its reliability, it presents an ICC between 0.85 and 0.91, evaluated in the Spanish population [31]. In HADS, each question is to be answered with one of four options, which describe perceived frequency. Questions with an odd number are related to anxiety, whereas even-numbered questions concern depression. The result of the addition of all scores determines how deeply the patient’s mood is affected, with higher scores revealing a larger effect [30]. This score was measured just before the beginning of the first treatment session and again after the end of the last one.

#### 2.3.4. Other measures

In order to assure the normal distribution of participants according to demographic data, measurements of age, weight, height and body mass index were carried out. The number of years since the participants were diagnosed of fibromyalgia was also considered, counting it as one more year if less than six months were left for a complete year.

### 2.4. Statistical Analysis

Description of the data was made using means and standard deviations for the continuous variables, and by frequencies and percentages for the categorical ones. Kolmogorov–Smirnov and Levene’s tests were used to test normal distribution of the sample and homoscedasticity of the groups, respectively. In order to test the comparability of groups at baseline, one-way analysis of variance (ANOVA) was used. To test the effect of the treatment, a linear mixed model was used. The hypothesis of interest was time-by-group interaction. Due to the between-group differences at baseline regarding the height of participants, the analysis was controlled using this as a covariable. 

Coefficient of determination (R^2^) was used to measure effect size (ES). According to Cohen, ES can be deemed insignificant if R^2^ <0.02, small if R^2^ is between 0.02 and 0.15, medium when R^2^ is between 0.15 and 0.35 and large when R^2^ >0.35 [32].

Management and data analysis were performed using the Statistical Package for the Social Sciences V.23 (IBM, Armonk, NY, USA). We worked with a 95% confidence level (*p* < 0.05).

## 3. Results

Sixty-six female participants agreed to participate in the study and completed the treatment and evaluation. Sociodemographic characteristics are shown in Table 1. Initially, the groups were comparable for most of the variables, but there were differences in height and BMI at baseline.

Analysis of covariance showed time-by-group interactions for global pain, local pain, and all the ICAF subscales except for passive coping. Effect size was large for local pain (R^2^ = 0.455), the total score of ICAF (R^2^ = 0.422) and PF-ICAF (R^2^ = 0.381) (Table 2).

Within-group change was not statistically significant for any variable in the control group, for total ICAF_T, ICAF_FF, or ICAF_FE in the sham group, or for most of the variables of the experimental group except HADS (Table 3).

Between-group pairwise comparison showed significant differences between the intervention and sham groups, but only for local pain and the ICAF physical factor score (Table 4).

Finally, no adverse events were reported by any patient.

## 4. Discussion

The aim of this study was to analyze the effectiveness of a non-invasive treatment that uses MDR to address the main symptoms of fibromyalgia. Findings show encouraging results concerning local pain reduction in the upper trapezius area and physical factor scores, which are linked to the quality of life of patients with fibromyalgia in the short term. Despite this, long-term effects remain unknown.

This study is the first clinical trial that analyzed the effects of the application of a monopolar dielectric radiofrequency electro-analgesic technique using the emission of electromagnetic signals in participants with fibromyalgia. This establishes a precedent regarding the design and methodology of further studies that may apply similar therapies. Moreover, and unlike the present work, many studies have not performed sham interventions to assess the effectiveness of electrotherapy devices versus placebo [33,34].

Based on the promising results achieved in this study, MDR therapy appears to be an effective way of treating local and general pain in patients with fibromyalgia in the short-term, as no side effects were observed and the pain reduction produced was important. While, with TENS, Carbonario et al. obtained a pain decrease of 23 mm in VAS [19] and Lauretti et al. obtained 25mm [35], MDR therapy in this study reached 38mm of pain reduction. Mutlu et al. studied the effects of TENS combined with exercise, obtaining poor effects on pain for TENS alone [34]. For other authors, was unclear whether TENS produces analgesic effects in fibromyalgia or not, as studies did not use placebo and the effects observed were poor [33,36]. It must also be remarked that most of the studies only assessed local pain [19,33], though effects of MDR in general pain reduction reached 18mm. For all the previous cases, MDR therapy may induce higher degrees of improvement than electrical nerve stimulation. To assure such a conclusion, further studies should compare MDR with TENS devices. Given that MDR therapy shares some analgesic mechanisms with TENS [18], the difference may be found in the type of monopolar dielectric transmission type, which depends on tissue load capacity instead of tissue conductivity. This may allow for transmitting a higher energy density than devices that apply resistive transmission [22]. 

The placebo effect in this type of therapies usually reaches important clinical levels [18,36], and for this reason using a sham group was both justified and necessary. The results obtained by the sham group in this study are noticeable, but far from the ones obtained by the experimental group concerning the local pain and physical factors related to quality of life, which did in fact reach statistical significance. This important placebo effect may be linked to the highly complex biopsychosocial impact that fibromyalgia patients experience [37], which increases the difficulty of obtaining accurate data because of their expectation of finding a cure for their symptoms. 

Regarding pharmacological treatment, some controversy exists about its effects on patients with fibromyalgia syndrome. Some studies support treatments using duloxetine, milnacipran, pregabalin, or amitriptyline [12,13,38]. Contrarily, some others do not recommend their use because of their potential for side effects and dependency [15,39]. According to the data of this study, a therapy using MDR yields similar results with far fewer side effects [12]. This may justify its inclusion as a complementary treatment.

With regard to quality of life, previous studies do not show any significant improvement when using TENS, nor other electrical stimulation treatments [19,36,40], yet another reason why MDR treatment seems encouraging. Exercise is accepted as the best option to improve quality of life and function in fibromyalgia [41] This approach has shown benefits in those quality of life factors more related to physical function, which can be explained by the relationship among pain, function and quality of life [42]. Pain reduces function and increases kinesiophobia in patients, which in the long term limits their capacity to be self-dependent, impacting their quality of life [42]. The analgesic effects of MDR in patients with fibromyalgia could facilitate a return to movement and physical exercise [43], which is a key point to improve patients’ quality of life [44]. For this reason, it could be recommended to add MDR to a physical exercise approach in patients with fibromyalgia. 

Quality of life factors related to patients’ coping did not shown differences with MDR treatment. These factors were not expected to change due to their relationship with patients’ behavior, as electrophysical agents seem to have no effects on the way patients cope with their symptoms in the short term [45]. Maybe longer treatments could lead to changes in patient daily behavior if they realize improvements in pain and function; however, this is unknown as only congnitive-behavioral therapy has shown effects on this [46].

No significant differences between sham and experimental groups were found as far as emotional functioning was concerned. However, other treatments such as manual therapy [47] and physical exercise [44,48] did have an influence on patient depression scores. This may be attributed to the therapeutic effects of human contact, which releases oxytocin, [49] and to the production of endorphins and the improvement in functionality levels brought about by physical activity [48,50]. None of these mechanisms are present in our MDR treatment, as this system is supposed to have no effects on the hypothalamic–pituitary–adrenal axis [2]. For this reason, additional benefits might have appeared if MDR had been complemented by manual therapy or physical exercise. 

We have failed to find any studies dealing with the efficacy of electrophysical treatment for improving quality of sleep in patients with fibromyalgia. However, improvements in the physical factor (a category which includes a quality-of-sleep item) have been observed in this study. Measuring quality of sleep independently from the physical factor would be interesting in future studies to assess the actual influence of MDR treatment on this outcome.

This study presents some limitations. Firstly, only short-term results were measured. As a result, follow-up studies will be required to observe the extent and duration of improvements in the long term. Secondly, caution should be exercised before extrapolating the effects of this study. This is a syndrome with a highly complex clinical profile and biopsychosocial impact, with suspected and extensive under-, over-, and misdiagnosing [51], all of which introduce a considerable risk of bias in any research. Thirdly, studies comparing MDR with other electrotherapy techniques are needed to find out which treatment is more useful for each specific symptom. Finally, finding out more about the analgesic effects of therapy with MDR when applied to other musculoskeletal impairments would be of high interest, as well as determining whether their effects are similar to those experienced by patients with fibromyalgia.

## 5. Conclusions

In the short term, MDR therapy relieves pain and improves quality of life related to the physical factor in participants with fibromyalgia. However, no significant between-group differences were found concerning participants’ mood or coping factors in quality of life. Thus, MDR could be recommended as a treatment for pain and function in fibromyalgia.

## Figures and Tables

**Table 1:** Sociodemographic characteristics of the sample and baseline comparability of the groups.

Outcomes	All		Control Group		Sham Group		Experimental Group		ANOVA
	Mean	SD	Mean	SD	Mean	SD	Mean	SD	*p*-Value
Age	49.00	7.17	50.63	6.69	47.78	6.32	48.57	8.25	0.461
Height	1.64	0.05	1.64	0.04	1.67	0.03	1.62	0.05	0.003
Weight	65.47	4.11	65.05	4.03	64.89	2.14	66.33	5.33	0.485
BMI	24.39	2.40	24.36	2.40	23.29	1.20	25.36	2.81	0.024
Years diagnosed	6.58	2.53	6.43	2.66	6.82	2.08	6.48	2.87	0.861
Pain_G	6.80	1.62	6.57	1.33	7.09	1.93	6.74	1.57	0.567
Pain_L	6.86	2.07	6.76	1.81	6.36	2.66	7.43	1.53	0.218
HADS	28.20	6.31	29.29	5.39	27.20	8.91	28.09	4.16	0.576
ICAF_T	20.99	10.18	18.72	11.83	22.98	8.00	21.16	10.45	0.395
ICAF_PF	60.52	11.78	55.86	14.74	64.55	8.08	60.91	10.64	0.050
ICAF_EF	23.42	10.40	20.71	12.17	25.18	7.26	24.22	11.13	0.340
ICAF_AC	67.64	26.89	61.52	25.86	72.64	21.59	68.43	31.96	0.399
ICAF_PC	37.70	15.43	35.10	17.08	40.91	12.05	37.00	16.79	0.456

SD: standard deviation; Pain_G: general pain; Pain_L: local pain at upper trapezious; ICAF_T: total ICAF score; ICAF_PF: ICAF Physical Factor; ICAF_EF: ICAF Emotional Factor; ICAF_AC: ICAF Active Coping; ICAF_PC: ICAF Passive Coping.

**Table 2:** Scores of the groups in the outcomes variables and results of the analysis of covariance using height as a covariable.

Variable	Group	Pre		Post				
		Mean	SD	Mean	SD	*p*-Value	R^2^	Power
Pain_G	Control	6.57	1.33	6.67	1.49	0.001	0.254	0.953
	Sham	7.09	1.93	6.64	1.87			
	Experimental	6.74	1.57	4.91	2.43			
Pain_L	Control	6.76	1.81	7.33	1.35	<0.001	0.455	1.000
	Sham	6.36	2.66	5.91	2.11			
	Experimental	7.43	1.53	3.61	2.62			
HADS	Control	29.29	5.39	32.35	5.49	0.456	0.032	0.179
	Sham	27.20	8.91	29.00	5.40			
	Experimental	28.09	4.16	31.91	4.69			
ICAF_T	Control	18.72	11.83	18.05	10.63	<0.001	0.422	1.000
	Sham	22.98	8.00	17.07	6.92			
	Experimental	21.16	10.45	9.61	11.05			
ICAF_PF	Control	55.86	14.74	56.33	14.21	<0.001	0.381	0.999
	Sham	64.55	8.08	53.82	12.34			
	Experimental	60.91	10.64	41.17	16.24			
ICAF_EF	Control	20.71	12.17	19.57	12.31	<0.001	0.284	0.976
	Sham	25.18	7.26	18.55	6.62			
	Experimental	24.22	11.13	14.17	8.54			
ICAF_AC	Control	61.52	25.86	60.43	20.23	0.032	0.131	0.651
	Sham	72.64	21.59	67.55	26.13			
	Experimental	68.43	31.96	77.39	25.32			
ICAF_PC	Control	35.10	17.08	31.81	17.20	0.076	0.100	0.513
	Sham	40.91	12.05	44.18	12.55			
	Experimental	37.00	16.79	29.65	17.22			

Pre: before treatment measures; Post: after treatment measures; SD: standard deviation; R^2^: effect size; Pain_G: general pain; Pain_L: local pain at upper trapezious; ICAF_T: total ICAF score; ICAF_PF: ICAF Physical Factor; ICAF_EF: ICAF Emotional Factor; ICAF_AC: ICAF Active Coping; ICAF_PC: ICAF Passive Coping.

**Table 3:** Within-group differences of the variables.

Outcomes	Control Group				Sham Group				Experimental Group			
	Mean	Lower	Upper	*p*-Value	Mean	Lower	Upper	*p*-Value	Mean	Lower	Upper	*p*-Value
Pain_G	0.26	−0.38	0.89	0.418	0.09	−0.70	0.88	0.818	1.81	1.20	2.42	<0.001
Pain_L	−0.46	−1.47	0.55	0.360	0.25	−1.00	1.50	0.693	3.76	2.79	4.72	<0.001
HADS	−3.29	−7.22	0.65	0.100	0.31	−4.56	5.19	0.898	−3.44	−7.21	0.33	0.073
ICAF_T	0.10	−2.92	3.13	0.947	5.88	2.13	9.62	0.003	12.40	9.51	15.30	<0.001
ICAF_PF	−0.82	−6.39	4.75	0.768	12.59	5.69	19.48	0.001	19.69	14.37	25.02	<0.001
ICAF_EF	0.84	−2.50	4.18	0.616	7.37	3.24	11.51	0.001	10.70	7.50	13.89	<0.001
ICAF_AC	3.08	−5.37	11.52	0.468	5.20	−5.26	15.67	0.322	−10.39	−18.48	−2.31	0.013
ICAF_PC	2.94	−2.75	8.63	0.304	−3.05	−10.09	4.00	0.389	7.75	2.31	13.19	0.006

Pain_G: general pain; Pain_L: local pain at upper trapezius; ICAF_T: total ICAF score; ICAF_PF: ICAF Physical Factor; ICAF_EF: ICAF Emotional Factor; ICAF_AC: ICAF Active Coping; ICAF_PC: ICAF Passive Coping; Lower: lower limit; Upper: upper limit.

**Table 4:** Between-group differences of the outcome variables.

Outcome	Experimental Group vs.	Mean Difference	Standard Error	95 % Confidence Interval for Mean Difference		*p*-Value
				Upper bound	Lower bound	
Pain_G	Control Group	1.617	0.662	−0.024	3.259	0.055
	Sham Group	1.720	0.798	−0.259	3.698	0.109
Pain_L	Control Group	3.659	0.677	1.982	5.337	<0.001
	Sham Group	2.242	0.816	0.220	4.263	0.025
HADS	Control Group	0.188	1.604	−3.788	4.164	1.000
	Sham Group	−4.720	1.933	−9.513	0.073	0.055
ICAF_T	Control Group	9.259	3.167	1.407	17.111	0.016
	Sham Group	8.170	3.818	−1.295	17.635	0.112
ICAF_PF	Control Group	15.196	4.345	4.426	25.966	0.003
	Sham Group	13.980	5.237	0.998	26.963	0.031
ICAF_EF	Control Group	5.992	3.159	−1.839	13.823	0.191
	Sham Group	5.031	3.808	−4.409	14.470	0.578
ICAF_AC	Control Group	−16.340	8.119	−36.466	3.785	0.149
	Sham Group	−5.932	9.786	−30.192	18.328	1.000
ICAF_PC	Control Group	4.265	5.327	−8.940	17.469	1.000
	Sham Group	13.451	6.421	−2.466	29.368	0.124

Pain_G: general pain; Pain_L: local pain at upper trapezious; ICAF_T: total ICAF score; ICAF_PF: ICAF; Physical Factor; ICAF_EF: ICAF Emotional Factor; ICAF_AC: ICAF Active Coping; ICAF_PC: ICAF Passive Coping.
